# Technical Variability Is Greater than Biological Variability in a Microarray Experiment but Both Are Outweighed by Changes Induced by Stimulation

**DOI:** 10.1371/journal.pone.0019556

**Published:** 2011-05-31

**Authors:** Penelope A. Bryant, Gordon K. Smyth, Roy Robins-Browne, Nigel Curtis

**Affiliations:** 1 Department of Paediatrics, The University of Melbourne, Melbourne, Australia; 2 Infection, Immunity and Environment Theme, Murdoch Children's Research Institute, Melbourne, Australia; 3 Infectious Diseases Unit, Department of General Medicine, Royal Children's Hospital, Melbourne, Australia; 4 Bioinformatics Division, Walter and Eliza Hall Institute of Medical Research, Melbourne, Australia; 5 Department of Mathematics and Statistics, The University of Melbourne, Melbourne, Australia; 6 Department of Microbiology and Immunology, The University of Melbourne, Melbourne, Australia; University of Minnesota, United States of America

## Abstract

**Introduction:**

A central issue in the design of microarray-based analysis of global gene expression is that variability resulting from experimental processes may obscure changes resulting from the effect being investigated. This study quantified the variability in gene expression at each level of a typical *in vitro* stimulation experiment using human peripheral blood mononuclear cells (PBMC). The primary objective was to determine the magnitude of biological and technical variability relative to the effect being investigated, namely gene expression changes resulting from stimulation with lipopolysaccharide (LPS).

**Methods and Results:**

Human PBMC were stimulated *in vitro* with LPS, with replication at 5 levels: 5 subjects each on 2 separate days with technical replication of LPS stimulation, amplification and hybridisation. RNA from samples stimulated with LPS and unstimulated samples were hybridised against common reference RNA on oligonucleotide microarrays. There was a closer correlation in gene expression between replicate hybridisations (0.86–0.93) than between different subjects (0.66–0.78). Deconstruction of the variability at each level of the experimental process showed that technical variability (standard deviation (SD) 0.16) was greater than biological variability (SD 0.06), although both were low (SD<0.1 for all individual components). There was variability in gene expression both at baseline and after stimulation with LPS and proportion of cell subsets in PBMC was likely partly responsible for this. However, gene expression changes after stimulation with LPS were much greater than the variability from any source, either individually or combined.

**Conclusions:**

Variability in gene expression was very low and likely to improve further as technical advances are made. The finding that stimulation with LPS has a markedly greater effect on gene expression than the degree of variability provides confidence that microarray-based studies can be used to detect changes in gene expression of biological interest in infectious diseases.

## Introduction

Microarrays provide a powerful tool to quantify global gene expression. A potential limitation is that variability resulting from experimental processes may obscure changes resulting from the effect being investigated. If the variability is substantial or systematic, it may be erroneously interpreted as a genuine difference. To date, there have been few studies quantifying the variability and reproducibility of microarray experiments in humans.

Sources of variability include biological (between subjects) and technical (everything downstream from obtaining an RNA sample) [1]. One study in humans assessing biological variability found gene expression was influenced by a variety of factors including age, sex, time of day of sampling and constituent cell subsets [2]. Further, there have been found to be familial similarities in variability in baseline gene expression [3], [4]. Technical variability could result from any of the multiple steps involved in the detection of gene expression changes using microarrays including amplification of RNA and hybridisation [5]. Previous *in vitro* microarray studies in tissue and cell lines to investigate whether technical or biological variability is greater have found inconsistent results. For example, one study investigating variance in gene expression in lymphoblastoid cells, found that for the majority of genes variance between individuals (biological) was greater than variance between replicates (technical) [3]. In another study there was a low degree of technical variability when comparing two samples of identical RNA prepared from a cell culture, but the results were not markedly different when different cell culture preparations were used, suggesting that the majority of the variability was technical [1]. A separate arm of the study showed that in some cases different cell lines from the same individual had higher variability than the same cell lines from different individuals. Different subjects, cell lines and technical steps were not all compared directly with each other and the component sources of variability from each step of the process were not deconstructed. The authors suggested that inter-individual differences might mask changes due to a stimulus.

The aim of this study was to investigate the variability in gene expression in an *in vitro* stimulation experiment using human peripheral blood mononuclear cells (PBMC). The primary objective was to determine the magnitude of biological and technical variability relative to the effect being investigated, namely gene expression changes resulting from stimulation with lipopolysaccharide (LPS). We aimed to identify the relative contribution of different sources of variability at each stage of the experiment culminating in hybridisation to a microarray slide. The results were intended to determine to what extent detected gene expression differences in an *in vitro* microarray experiment in humans can be attributed to the stimulus investigated rather than artefactual differences from technical and biological variation.

## Results

**Figure 1:**
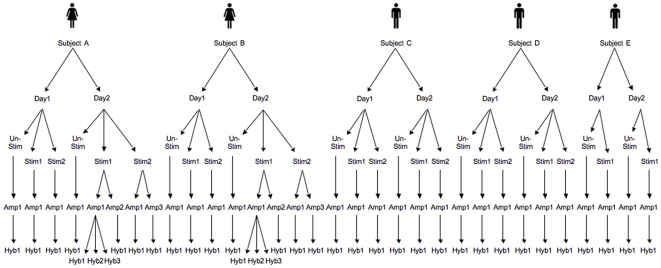
Experimental design showing samples used to compare variability. Samples: Subject A–E, Day 1 and 2, LPS-stimulated sample (Stim) 1 and 2, amplification run (Amp) 1–3, and hybridisation (Hyb) 1–3. For each subject on each day there was also an unstimulated (Unstim) sample at 0 hours.

An *in vitro* stimulation experiment was undertaken with replication at 5 levels (subject, day, stimulation tube, amplification and hybridisation) (figure 1). Five subjects each had blood taken on 2 days and their peripheral blood mononuclear cells (PBMC) were separated and stimulated with LPS in 2 parallel tubes for 24 hours or left unstimulated. All samples were amplified and RNA was hybridised against common reference RNA from pooled unstimulated samples from all subjects. Replication was also included at the amplification and hybridisation steps. Differences in gene expression with LPS stimulation were measured by comparing the log_2_ ratio of the stimulated samples with the unstimulated samples. Variability in gene expression was compared between all samples at baseline and after LPS stimulation.

### Cumulative effects of variability at different levels

**Figure 2:**
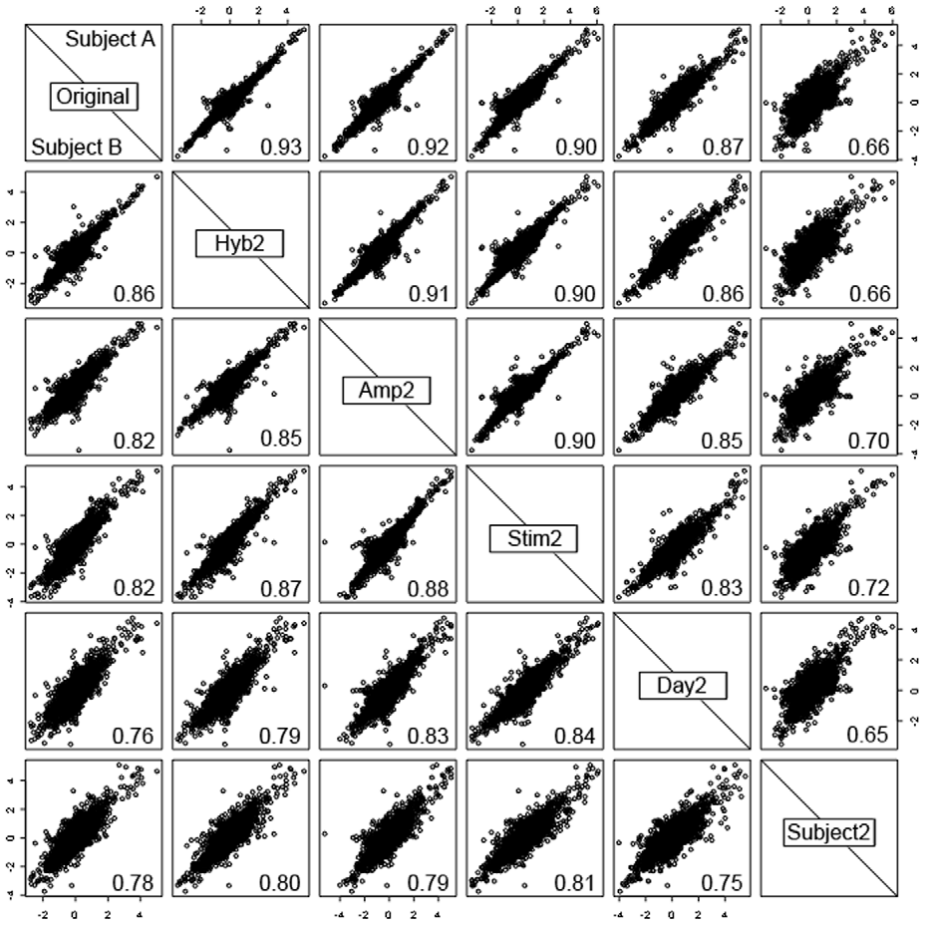
Matrix of scatterplots showing log_2_ ratios for replicate hybridisations at each level of the experiment. The fold changes for each sample are plotted against those for each of the other samples for gene expression after stimulation with LPS for two subjects: subject A (top right of diagonal) and subject B (bottom left). Comparisons are made at different levels of the experiment: hybridisation (Hyb2), amplification (Amp2), stimulation (Stim2), day (Day2) and subject (Subject2). Correlation coefficients between each pair of samples are shown in the bottom right of each box.

The hierarchy of levels of variability which underlie the experimental design are shown by comparing gene expression for the 2 subjects (A and B) who had replication at every level of the experiment (figure 2). Comparisons were made between gene expression (against the common reference) from one hybridisation and gene expression from hybridisations at each different level of replication. For replication at the subject level, one of the other subjects on the same day was randomly selected for comparison.

In general, for both subjects, hybridisations correlated more closely the further down the experimental process the replication occurred; for example, replication by hybridising the same RNA (Hyb2) had a correlation coefficient of 0.86–0.93, while hybridising RNA from 2 different subjects (Subject2) had a correlation of 0.66–0.78. This is because the variance is additive; for example, the variance (σ^2^) at the level of amplification is a combination (σ^2^_Amp_+σ^2^_Hyb_) of the variability due to amplification (σ^2^_Amp_) and the variability due to hybridisation (σ^2^_Hyb_).

### Multi-level random effects

To deconstruct this and to determine how much variability there was at each level of the experiment including the LPS interaction with subject and day (‘Subject:LPS’ and ‘Day:LPS’), a multi-level mixed model analysis was undertaken, incorporating both fixed and random effects (table 1) [6].

**Table 1:** Different components contributing to variability in the design of the study.

Component	Variability	Effect
Sex	Biological	Fixed
Subject	Biological	Random
Day	Biological	Random
LPS treatment	Physiological	Fixed
Stimulation tube	Technical	Random
Amplification	Technical	Random
Hybridisation	Technical	Random

The variance at each level was in principle estimated by subtracting the variance of the layers below it. Thus in the example above, by subtracting the variance at the level of hybridisation (σ^2^_Hyb_) from the combined variance at the level of amplification (σ^2^_Amp_+σ^2^_Hyb_), the variability due to amplification alone (σ^2^_Amp_) can be estimated.

**Figure 3:**
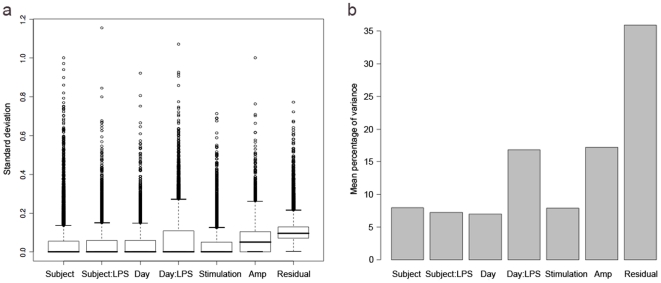
Variability of gene expression for each component level of the experiment. a) Standard deviation of all genes for each variance component, showing median standard deviation, interquartile ranges and outliers; b) Proportion of variance contributed by each component to the overall variance. Amp - amplification run.

The median standard deviation (SD, σ) for all of the variance components was very small, at less than 0.1 for all components, and 0 for the majority (figure 3a). Note that ‘Residual’ refers to the variance component attributable to hybridisation, but also includes any other factors contributing to variability downstream of amplification. The greatest variability was at the levels of amplification and hybridisation, but even these were small. The proportion that each component contributed to the overall variance indicated that the greatest proportion of variance was contributed by Residual/Hybridisation, which was double that contributed by Amplification, and nearly four times that contributed by most of the remaining components (figure 3b). Although the variance between different days was slightly smaller than between different subjects, when the LPS interaction was taken into account, the interaction between Day and LPS contributed variability greater than the Subject or Subject:LPS interaction.

**Figure 4:**
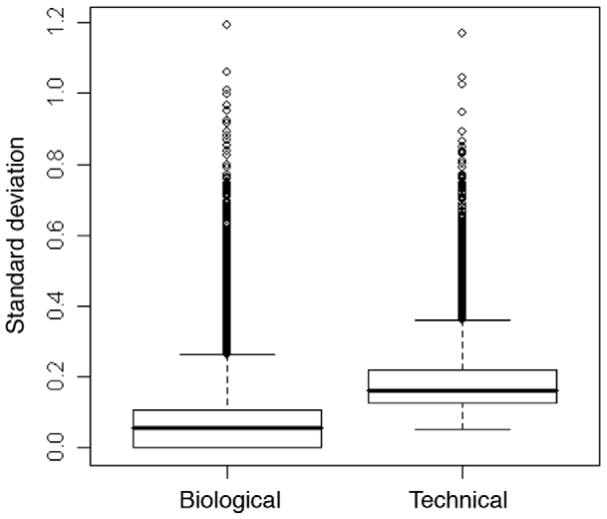
Standard deviation of all genes for biological and technical variance, showing median standard deviation, interquartile ranges and outliers.

A comparison was made between biological and technical variability by summing the Subject and Day components for biological variability, and the Stimulation, Amplification and Residual components for technical variability (figure 4). Technical variability (SD 0.16) was slightly greater than biological variability (SD 0.06), although they were both low with the upper interquartile range of the standard deviation for each being less than 0.4.

Comparing combined biological and technical variance also shows that the variance did not greatly increase when adding different variance components together. Although the components contributing to the variability are additive, the same genes were not highly variable at each level, so the overall variability was not markedly higher.

### LPS response between individuals

The LPS effect is the difference between stimulated and unstimulated samples. Overall the inter-subject variability in response to LPS was very low with the median standard deviation at zero (figure 3a). Although the majority of genes showed consistent expression, there were a few genes that varied between subjects in response to LPS. Genes were ranked by their Subject:LPS variance component, and the top 10 genes that were most variable between subjects in terms of their response to LPS are shown in table 2.

**Table 2:** The top 10 most variable genes between subjects by stimulation with LPS and the standard deviation (SD) of each.

Accession no.	Gene name	Gene symbol	SD
NM_002421	Matrix metalloproteinase 1	MMP1	1.16
NM_006770	Macrophage receptor with collagenous structure	MARCO	0.85
NM_001925	Defensin, alpha 4, corticostatin	DEFA4	0.80
NM_004131	Granzyme B	GZMB	0.68
X62468	Interferon gamma	IFNG	0.67
NM_000963	Prostaglandin-endoperoxide synthase 2	PTGS2	0.67
NM_002964	S100 calcium-binding protein A8	S100A8	0.65
NM_004244	CD163 antigen	CD163	0.64
NM_006850	Interleukin 24	IL24	0.62
NM_007115	Tumor necrosis factor, alpha-induced protein 6	TNFAIP6	0.59

The majority of these genes are recognisable as being involved in the immune response, with a variety of cytokines and receptors represented. This is consistent with previous findings of differences in *in vitro* cytokine responses to stimulation with LPS between individuals [7], [8]. Potential causes of this subset of genes showing variable response to LPS between subjects were further investigated. Baseline gene expression in peripheral blood from human subjects has previously been found to be affected by age, sex, time of day of sampling and constituent cell subsets [2]. In animals the response to LPS is also affected by age and sex [9]. In this study the subjects had a narrow age range and blood was taken at the same time each day so these variables were excluded, and the effect of sex and constituent cell subsets was further analysed.

### Effect of sex on LPS response

**Figure 5:**
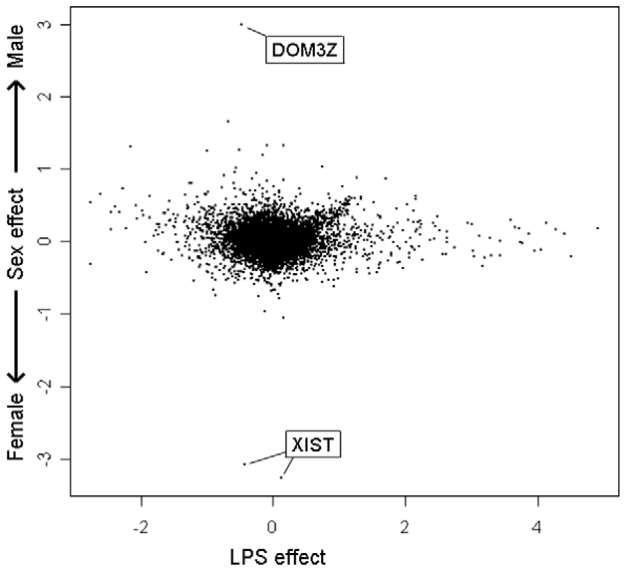
Effect of LPS and sex on gene expression with log2 ratios contributed by each component along the x and y axes respectively.

There is almost no overlap between genes that respond to LPS and genes that are expressed differently between the sexes (figure 5). The genes most differentially expressed between the sexes were XIST (represented by 2 spots on the microarray) and DOM3Z (figure 5). XIST is a gene expressed only on the inactivated X chromosome, therefore only in females [10]. DOM3Z is a gene found in the MHC III region paired with RP1, a protein thought to be involved in androgen-responsiveness in male mice [11] which could explain its sex effect. However, the effect of LPS on their expression was minimal. Although not designed to investigate the effects of sex on LPS response, further analysis showed that this effect was not strong in this study.

### Effect of different days and cell subset proportions

Although the median standard deviation for both was zero, the variability in response to LPS between days was unexpectedly greater than between subjects (figure 3b). All hybridisations were with the common reference RNA: from unstimulated PBMC from subjects A–D on Day 2. There was therefore less differential expression between the Day 2 samples and the common reference than between the Day 1 samples and the common reference. This suggests the finding is at least in part due to artefact.

The prime effector cells following LPS stimulation are monocytes (CD14+), and there were differences in the proportion of monocytes in PBMC samples from each subject on each day (table 3). The greatest difference in proportion of monocytes between the two days was 4.8% at 0 hours (subject B) and 7.5% at 24 hours (subject D). This difference in cell subset proportions may partly explain the difference between Day 1 and Day 2. However, the day-to-day variability in monocyte percentages is less than that between subjects, so monocyte percentage alone does not to explain the relatively high Day:LPS variance component.

**Table 3:** Proportion of monocytes (CD14+) in different samples.

	CD14+ cells (%)			
Subject	0 h		24 h	
	Day 1	Day 2	Day 1	Day 2
**A**	7.7	7.1	6.4	6.2
**B**	2.3	7.1	3.5	3.2
**C**	5.9	8.4	8.2	11.0
**D**	11.9	14.6	7.0	14.5
**E**	1.8	2.8	1.1	1.6

There was a wide range of monocyte proportions in PBMC between subjects at 0 and 24 hours and some of the most variable genes are known to be strongly expressed in monocytes, for example, MMP1, CD163, MARCO and IL-24 [12], [13], [14]. There were also genes amongst the most variable known to be expressed in other leukocytes, for example IFNG (T cells) [15] and GZMB (cytotoxic T cells) [16]. Although they were not measured, these cell subset proportions also likely differed between subjects.

### Variability compared to biological effect

**Figure 6:**
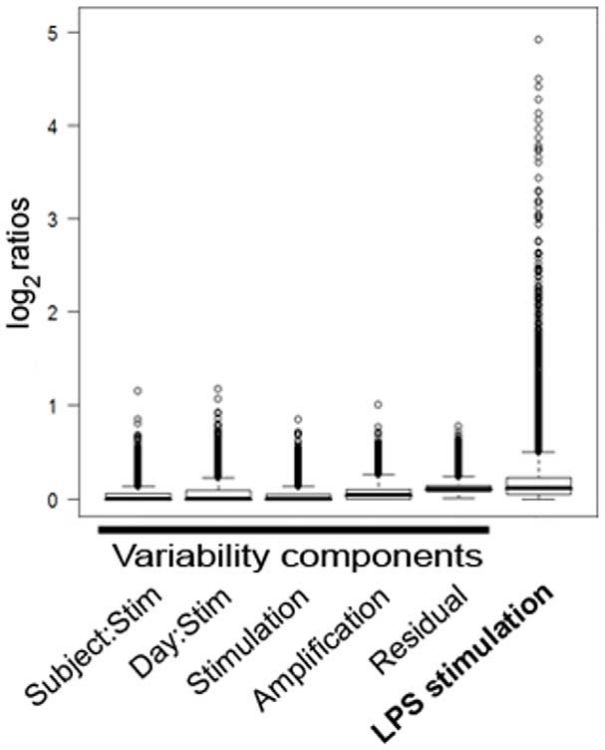
Standard deviations of the different variability components and gene expression values after LPS stimulation on the same log_2_ scale, showing median standard deviation, interquartile ranges and outliers.

When comparing LPS-stimulated to unstimulated samples, 4552 genes were significantly differentially expressed. A comparison of the fold changes after LPS stimulation with the variability expressed as a standard deviation on the same scale shows gene expression in response to LPS dwarfs biological or technical variability (figure 6).

## Discussion

Technical variability is inherent in all microarray experiments as a result of the number of elements being measured and the number of steps in the process that culminates in hybridisation of RNA to a microarray slide. Different methods have been used to attempt to control the variability in microarray experiments, including the use of control housekeeping genes on the array [17], pre-processing and normalisation of data [18], [19], [20] and replication [21] which allows an estimation of error. In recent studies, variability has even been exploited to enhance microarray data analysis [22] and calculate sample size [23]. For these experimental and bioinformatic methods to be relevant to biology, an understanding of the sources and magnitude of variability in gene expression is crucial.

This is the first study to deconstruct the variability in a microarray experiment into each level of the experimental process. All sources of variability in this study were low. Even the largest source of variability, the residual component measured by replicate hybridisations, had a standard deviation of about 0.1 on the log_2_ scale, corresponding to 7% of gene expression level. The low variability was partly a result of using high quality normalization and pre-processing. Less attention to issues such as background correction would have resulted in higher variability [18]. However, large scale filtering of spots on quality grounds was not undertaken, and filtering was used only to remove transcripts not expressed in peripheral blood.

The minimal variability found between subjects in gene expression in peripheral blood is consistent with other human gene expression studies. Whitney *et al* showed low variability in baseline gene expression [2]. Our study confirmed this and additionally is the first to show that there is low variability in response to LPS stimulation. The response to LPS is known to be highly conserved, even between species [24], and it is possible that there would be more variability in gene expression in response to a different stimulant where the response was less stereotypical. Differences between subjects in response to LPS related largely to genes involved in the immune response, which may explain why individuals have different clinical responses to the same organism and why some individuals have poor outcomes with sepsis. In animals, sex, pregnancy, age and stress have all been shown to affect the response to LPS [9], [25], [26]. A key finding was that the differential expression induced by LPS was markedly greater than any differences resulting from technical or biological variability. This was true of each of the deconstructed components contributing to variability, and when individual sources were combined.

The largest sources of variability in this experiment were the technical steps of amplification and hybridisation. These steps can potentially be improved by technological developments as been showed by comparisons between different platforms [27].

Day to day variability was comparable to or greater than variation between subjects. The most likely explanation was that the finding was an artefact due to the composition of the RNA contributing to the common reference. As long as it is common to all hybridisations, the composition of the reference RNA has previously been thought not to matter. However, because the differential expression was not identically distributed between Days 1 and 2 compared to the common reference, Day 2 had a much smaller variance. Comparison of Day 1 and Day 2 was therefore not random, and this likely created the artefact of there appearing to be a larger difference between days than subjects. However, while this finding may be partly artefactual, it is also consistent with the findings that samples from subjects who contributed blood on multiple days do not necessarily cluster together [2]. Factors such as how much sleep each subject got the night before, what they had for breakfast and whether they cycled or drove to work could all potentially affect gene expression responses [28], [29], [30]. This highlights the similarity between subjects in gene expression, both at baseline, and in response to LPS. This suggests that blood samples collected over multiple days may be preferable when investigating individual responses.

This study is the first to correlate variability with cell subset proportions before and after stimulation with LPS. Monocytes account for the largest proportion of cells in PBMC that respond to LPS. Genes not expressed in non-monocyte cells in response to LPS are likely to show a lower fold change overall in PBMC samples with smaller proportions of monocytes [31]. Hence monocyte proportion is likely to be a factor in variation, and measuring expression in purified cell populations may reduce variability further. However, the inter-subject variability was very low in this study so differences in cell subset proportions did not have a strong effect.

The microarray quality control (MAQC) project [32] was designed to investigate variability in microarray experiments, but differs from this study in that its aim was to assess the quality of microarray technologies and therefore only investigated technical replication by hybridizing identical RNA samples. It did not aim to investigate biological replication and did not attempt to deconstruct the components of technical variation. Therefore, the results of this study are complementary to the MAQC project rather than being directly comparable to it.

In summary, we found that the variability attributable to technical and biological variation in a typical *in vitro* microarray experiment in humans is low, and markedly less than the effect on gene expression of stimulation. This provides confidence that microarray-based studies can be used to detect changes in gene expression of biological interest in infectious diseases.

## Methods

### Ethics statement

This study received approval from the Human Research Ethics Committee (23096A) at the Royal Children's Hospital, and informed consent was obtained verbally from the adult volunteers.

### PBMC separation and stimulation

Five adult volunteers (two female and three male, age range 21–34 years) had blood sampled on the same two days, one week apart, between 9 and 10 am (figure 3). Blood was collected into tubes containing endotoxin-free lithium heparin (Becton Dickinson, Franklin Lakes, NJ, USA). PBMC were separated by Ficoll-Hypaque gradient (Amersham Biosciences, Uppsala, Sweden). Aliquots of 2×10^6^ PBMC were simultaneously stimulated with 1 µg/ml LPS (Sigma Aldrich, Sydney, NSW, Australia) and incubated at 37°C with 5% CO_2_ for 0 and 24 hours. Each stimulation condition was undertaken in duplicate to enable comparison between two parallel stimulations of PBMC. At each time point, after centrifugation, TRIzol® (Invitrogen Life Technologies, Invitrogen Corporation, Carlsbad, CA, USA) was added to the sample before storing at −80°C. In addition, four samples were divided in half and each half was amplified on a different day.

### RNA preparation

RNA from all samples was extracted using the chloroform:phenol method within one month after stimulation before further storage at −80°C. Samples were then purified using the RNeasy™ kit (Qiagen Pty Ltd, Clifton Hill, VIC, Australia) and amplified using the MessageAmp™ II aRNA Kit (Ambion Inc, Austin, TX, USA) following the manufacturers' protocols. All samples were analysed post-amplification using an Agilent 2100 Bioanalyser (Agilent Technologies, Forest Hill, VIC, Australia). All RNA samples were of satisfactory quality.

### Cell population analysis by flow cytometry

For each subject on each day at each time point, 5×10^5^ PBMC were stained with PE-conjugated CD14 (IOTest®, Immunotech, Marseille, France) and 5×10^5^ cells were stained with PE-conjugated mouse IgG_1_ as a negative isotype control Cells were incubated in phosphate buffered saline (PBS), 0.1% sodium azide and 20 µl of the conjugated antibody at room temperature for 15 min, washed and resuspended in 300 µl PBS with 2% formalin. Analysis was undertaken using a LSR II flow cytometer and BD FACSDiva® software (Becton Dickinson).

### Microarray hybridisation

The study used 36 spotted microarrays printed with the Compugen human 19,000 oligonucleotide library (http://www.cgen.com) and a selection of control probes at the Adelaide Microarray Facility (Adelaide, Australia). To minimize variability, all microarrays were from the same printing batch, except 2 slides (Hyb3 from subjects A and B). There were negligible differences between slides from the different batches. Amplified RNA (aRNA) was labelled by a direct platinum-based labelling technique using a kit (ULS™ aRNA labelling, Kreatech Biotechnology, Amsterdam, The Netherlands) following the manufacturer's protocol. Each sample of experimental RNA (unstimulated and from each stimulation) was competitively hybridised with a common reference sample, obtained from pooling unstimulated RNA from subjects A–D from Day 2. For each pair of RNA samples to be hybridised to a slide, 2 µg of the pooled reference RNA was labelled with ULS™-Cy3 and 2 µg of the individual sample RNA with ULS™-Cy5. The samples amplified on a different day were also hybridised against the pooled RNA.

In addition, three hybridisations were undertaken using the same sample of RNA. This was done with two samples of RNA to provide replication. The two dye-coupled samples for each array were combined and fragmented using 4 µl Fragmentation Reagents (Ambion). The labelled sample was mixed with 10 µl 1 mg/ml human Cot-1 DNA (Invitrogen), 15 µl 20×SSC, 20 µl deionised formamide (Sigma Aldrich), 20 µl Kreatech solution (Kreatech Biotechnology) and 5 µl 10% SDS, heated at 95°C for 5 min and cooled to room temperature. Each sample was applied to a slide which was incubated in a water bath in the dark at 42°C for 18 hours, washed and scanned using a Genepix® 4000B scanner (Molecular Devices, Sunnyvale, CA, USA).

### Microarray data normalisation and analysis

Each scanned TIF image was quantified using Genepix Pro 6.0 software (Molecular Devices) to obtain foreground and background intensity values for each spot. Genepix was configured to generate the custom morphological close-open background estimator, which is less variable than the more usual local background estimators [33]. Pre-processing and quality assessment was done using the limma software package [34] for the R/Bioconductor programming environment (http://www.bioconductor.org). A small offset of 50 was added to the intensities after background correction to ensure that there were no negative background-corrected intensities or missing log-ratios, and to ensure that low-intensity log-ratios remained of low variability. Microarray data quality was checked using diagnostic image plots, MA-plots and control probes and was found to be satisfactory. Low-intensity probes were filtered on the basis of mean A-values, which give the average log_2_ intensity for each probe across all arrays. Log-ratios were print-tip loess normalised with span = 0.4, giving zero weight to probes with mean A-value<6.5 [19]. After normalization, control probes were removed from the data leaving only the Compugen library probes. To remove probes corresponding to transcripts not expressed in PBMCs, 33% of library probes with lowest mean A-values were filtered before subsequent analysis.

A linear model approach was used to analyse all the microarrays for the five individuals together. A multi-level mixed linear model was fitted to the normalized log-ratio expression data for each probe using the lmer() function in lme4 package for R [6]. The multi-level variance components were estimated by restricted maximum likelihood (REML) and the fixed effects were estimated by generalised least squares. The principle underlying the estimation of variance at each level of the experiment (by maximizing the REML likelihood) was the subtraction of the variance at each level below it. For example, the variability (σ^2^) between technical replication at the level of amplification is the sum of the variability at the level of amplification and the variability at the level of hybridisation (σ^2^_Amp_+σ^2^_Hyb_). Therefore by determining the variability introduced at the lowest level, hybridisation (σ^2^_Hyb_), the variability introduced at the level of amplification can be calculated ((σ^2^_Amp_+σ^2^_Hyb_)−(σ^2^_Hyb_) = σ^2^_Amp_). Similar calculations provide the variability at each level higher.

The mixed linear model included fixed effects for sex and LPS treatment, and random effects for each level of variability in the experimental design (figure 1, table 1). The model can be represented by the formula:
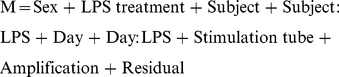
where M is the log_2_ expression value of a gene from an individual microarray slide. The mixed model analysis deconstructed the overall variability into the variability attributable to each of the different components. ‘Subject:LPS’ and ‘Day:LPS’ refer to the interaction between the fixed effect of stimulation with LPS and the variables of subject and day respectively (see section on LPS effect). ‘Residual’ is the variance component attributable to hybridisation, but also includes any other factors contributing to variability downstream of amplification.

To determine the variability with LPS stimulation, the interaction between LPS and different subjects and different days (designated Subject:LPS and Day:LPS respectively) was included in the model. The variance of M_LPS_ was determined by adding the measurement error or variance (σ^2^) for each of the variance components including the Residual component:
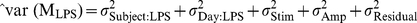
The Compugen-supplied GenBank accession numbers were mapped to gene symbols using SOURCE [35] and the UniGene build of 2nd September 2006. The study is MIAME compliant.
